# Paraimmunobiotic Bifidobacteria Modulate the Expression Patterns of Peptidoglycan Recognition Proteins in Porcine Intestinal Epitheliocytes and Antigen Presenting Cells

**DOI:** 10.3390/cells8080891

**Published:** 2019-08-14

**Authors:** Hikaru Iida, Masanori Tohno, Md. Aminul Islam, Nana Sato, Hisakazu Kobayashi, Leonardo Albarracin, AKM Humayun Kober, Wakako Ikeda-Ohtsubo, Yoshihito Suda, Hisashi Aso, Tomonori Nochi, Ayako Miyazaki, Hirohide Uenishi, Noriyuki Iwabuchi, Jin-zhong Xiao, Julio Villena, Haruki Kitazawa

**Affiliations:** 1 Food and Feed Immunology Group, Laboratory of Animal Products Chemistry, Graduate School of Agricultural Science, Tohoku University, Sendai 980-8572, Japan; 2 Livestock Immunology Unit, International Education and Research Center for Food Agricultural Immunology (CFAI), Graduate School of Agricultural Science, Tohoku University, Sendai 980-8572, Japan; 3 Central Region Agricultural Research Centre, National Agriculture and Food Research Organization, Nasushiobara 29-2793, Japan; 4 Department of Medicine, Faculty of Veterinary Science, Bangladesh Agricultural University, Mymensingh 2202, Bangladesh; 5 Laboratory of Immunobiotechnology, Reference Centre for Lactobacilli, (CERELA-CONICET), Tucuman 4000, Argentina; 6 Department of Food, Agriculture and Environment, Miyagi University, Sendai 980-8572, Japan; 7 Cell Biology Laboratory, Graduate School of Agricultural Science, Tohoku University, Sendai 980-8572, Japan; 8 Infection Immunology Unit, International Education and Research Center for Food Agricultural Immunology (CFAI), Graduate School of Agricultural Science, Tohoku University, Sendai 980-8572, Japan; 9 Viral Diseases and Epidemiology Research Division, National Institute of Animal Health, NARO, Tsukuba 305-0853, Japan; 10 Animal Bioregulation Unit, Division of Animal Sciences, Institute of Agrobiological Sciences, National Agriculture and Food Research Organization (NARO), Tsukuba, Ibaraki 305-8634, Japan; 11 Food Science and Technology Institute, Morinaga Milk Industry Co. Ltd., Zama, Kanagawa 252-8583, Japan; 12 Next Generation Science Institute, Morinaga Milk Industry Co. Ltd., Zama, Kanagawa 252-8583, Japan

**Keywords:** paraimmunobiotics, peptidoglycan recognition protein, bifidobacteria, innate immunity, porcine intestinal epitheliocytes, porcine antigen-presenting cells

## Abstract

Peptidoglycan recognition proteins (PGLYRPs) are a family of pattern recognition receptors (PRRs) that are able to induce innate immune responses through their binding to peptidoglycan (PGN), lipopolysaccharide, or lipoteichoic acid, or by interacting with other PRR-ligands. Recently, progress has been made in understanding the immunobiology of PGLYRPs in human and mice, however, their functions in livestock animals have been less explored. In this study, we characterized the expression patterns of PGLYRPs in porcine intestinal epithelial (PIE) cells and antigen-presenting cells (APCs) and their modulation by the interactions of host cells with PRR-ligands and non-viable immunomodulatory probiotics referred to as paraimmunobiotics. We demonstrated that PGLYRP-1, -2, -3, and -4 are expressed in PIE cells and APCs from Peyer’s patches, being PGLYPR-3 and -4 levels higher than PGLYRP-1 and -2. We also showed that PGLYRPs expression in APCs and PIE cells can be modulated by different PRR agonists. By using knockdown PIE cells for TLR2, TLR4, NOD1, and NOD2, or the four PGLYRPs, we demonstrated that PGLYRPs expressions would be required for activation and functioning of TLR2, TLR4, NOD1, and NOD2 in porcine epitheliocytes, but PGLYRPs activation would be independent of those PRR expressions. Importantly, we reported for the first time that PGLYRPs expression can be differentially modulated by paraimmunobiotic bifidobacteria in a strain-dependent manner. These results provide evidence for the use of paraimmunobiotic bifidobacteria as an alternative for the improvement of resistance to intestinal infections or as therapeutic tools for the reduction of the severity of inflammatory damage in diseases in which a role of PGLYRPs-microbe interaction has been demonstrated.

## 1. Introduction

The innate immune system provides the first line of defense against invading microorganisms employing different strategies to discriminate non-self-structures from self-molecules. This function is mediated by germ-line encoding pattern recognition molecules called pattern recognition receptors (PRRs) that recognize the conserved pathogen-associated molecular patterns (PAMPs) present in microorganisms but absent in the host, such as lipopolysaccharides (LPS) of Gram-negative bacteria, lipoteichoic acids (LTA) of Gram-positive bacteria or peptidoglycan (PGN) of both Gram-positive and -negative bacteria [1]. Peptidoglycan (PGN) is a peptide-cross-linked sugar polymer that is an essential cell wall component of virtually all bacteria [2]. Although the overall structure of PGN from different bacteria is similar, there are backbone and crosslinking modifications that increase the diversity among bacterial species. The innate immune system is able to sense intact PGN as well as PGN fragments using numerous PRRs that are secreted, expressed intracellularly or located on the cells’ surface. PGN and its fragments are primarily recognized by peptidoglycan recognition proteins (PGLYRPs or PGRPs), a novel family of PRR, which were initially named according to their ability to bind PGN [3,4]. In addition, PGLYRPs can also sense the ligands of other PRRs including toll-like receptors (TLRs) and nucleotide-binding oligomerization domain (NOD)-containing proteins [2,5]. 

Structurally all PGLYRPs have at least one C-terminal PGLYRP domain, which enables the interaction with bacterial PGN, and is homologous to the bacteriophage and to the prokaryotic PGN-lytic type 2 amidases [6,7]. PGLYRPs are conserved from insect to mammals and at least four members (PGLYRP-1, PGLYRP-2, PGLYRP-3, and PGLYRP-4) with distinct expression patterns have been identified in humans [3], mice [8], rats [9], and cattle [10]. Mammalian PGLYRP-1 is highly expressed in neutrophil’s granules, intestinal M cells [1], and also expressed as a serum dimeric protein [11]. PGLYRP-2 is an N-acetyle-muramoyl-L-alanine amidase, which can cleave the peptide from the glycan chain of PGN and is constitutively expressed in the liver, intestinal epithelial cells (IECs) and secreted into blood [12]. Studies in humans demonstrated that PGLYRP-3 and PGLYRP-4 proteins are selectively expressed in tissues that come in contact with the environment including the skin epidermis, sebaceous glands, the corneal epithelium of the eye, and the mucus-secreting cells of the main salivary gland [13]. PGLYRP-3 and PGLYRP-4 have also been detected in epithelial cells of the gastrointestinal tract of humans [9] and mice [7]. Despite the fact that pigs are important livestock animals and are also considered the most approximate animal model for human diseases, the expressions of PGLYRPs in porcine tissues and cells has been less explored. Therefore, the knowledge of the location where porcine PGLYRPs are expressed and stored, and how they are activated and regulated are important points of research. 

Previous studies have reported the antibacterial and immunomodulatory properties of human and mouse PGLYRPs and their expression in the gastrointestinal tissues [14,15]. PGLYPRs have evolved a variety of mechanisms to control the host’s mucosal interactions with mutualistic, commensal and pathogenic microorganisms to benefit the host [16,17]. Moreover, it was reported that mammalian PGLYRPs influence host-microbial interactions through their peptidoglycan-hydrolytic, bactericidal, and immunomodulatory properties [5,18]. Deficiencies in individual PGLYRPs can cause significant, but not identical, changes in gut normal microbiota and mucosal inflammatory responses [19,20]. 

In the past decade, the study of the cellular and molecular interactions of the intestinal microbiota members with epithelial and immune cells has clearly demonstrated the influence of those microorganisms on the host’s homeostasis [21]. Numerous benefits conferred by the microbiota have been characterized and in addition, bacterial strains with particular beneficial abilities have been selected for the development of products that are able to improve the health of the host. In this regard, probiotic bacteria have been proven to be efficient for ameliorating intestinal inflammation, improving epithelial barrier function, stimulating the mucosal immune system and preventing pathogenic microbial growth and colonization [22,23,24,25,26,27,28].

Members of the genus *Bifidobacterium* are among the first microbes to colonize the human gastrointestinal tract and are believed to exert positive health benefits on their host [23]. Several studies demonstrated that *bifidobacteria strains*, including *Bifidobacterium longum* subsp. *longum* BB536 and *Bifidobacterium breve* M-16V, as well as non-viable immunomodulatory bifidobacteria referred to as paraimmunobiotic bifidobacteria, are able to improve the resistance against respiratory and intestinal infections [24,25] and to reduce the severity of symptoms in inflammatory-mediated diseases [26,27,28]. Although some advances have been made in the understanding of the cellular and molecular interactions between paraimmunobiotic bifidobacteria with the host [29], their specific role in the regulation of PGLYRPs expression has not been explored.

In this work, we demonstrated that four PGLYRPs (PGLYRP-1, PGLYRP-2, PGLYRP-3, and PGLYRP-4) are expressed in the gastrointestinal tissues of pigs, especially in IECs and antigen-presenting cells (APCs). We showed that porcine PGLYRPs expression in APCs and IECs can be modulated by interactions in different PRR agonists. Importantly, we demonstrated for the first time that PGLYRPs expression in porcine APCs and IECs could be differentially modulated by paraimmunobiotic bifidobacteria, which sheds the light on immunobiotic mediated health benefits.

## 2. Materials and Methods

### 2.1. Ethics Statements, Collection, and Preparation of Tissue Samples

The study was carried out in strict accordance with the recommendations in the Guide for the Care and Use of Laboratory Animals of the Guidelines for Animal Experimentation of Tohoku University, Sendai, Japan. The present study was approved by the Animal Research and Animal Care Committee of the Tohoku University (2013 Noudou-017, 6th March 2013) and all efforts were made to minimize suffering.

Porcine tissues (spleen, mesenteric lymphoid nodes, and Peyer´s patches (PPs) from ileum and jejunum) were obtained from healthy adult LWD swine (*n* = 16; genotype 1/4 Landrace, 1/4 Large White, 1/2 Duroc) provided by the Miyagi Prefecture Animal Husbandry (Miyagi, Japan). Tissue sections were cut into 3 × 3 mm squares and treated with 1 mL of RNAlater^®^ Stabilization Solution (ThermoFisher Scientific, Chicago, IL, USA) and were transferred into round bottom propylene tubes (Falcon 2006, Becton Dickinson, Lincoln, NJ, USA) containing 1 mL of TRIzol (Invitrogen, Carlsbad, CA, USA) and stored at −80 °C. 

### 2.2. Gene Expression Analysis 

Total RNA was isolated using TRIzol reagent (Invitrogen, Carlsbad, CA, USA) and treated with gDNA Wipeout Buffer (Qiagen, Tokyo, Japan). All cDNAs were synthesized using a Quantitect reverse transcription (RT) kit (Qiagen, Tokyo, Japan), according to the manufacturer’s recommendations. Real-time quantitative PCR was carried out using a 7300 real-time PCR system (Applied Biosystems, Warrington, UK). The qRT-PCR was performed using a 7300 real-time PCR system (Applied Biosystems, Warrington, UK) and the TaqMan^®^ gene expression assay kit (Life Technologies, New York, NY, USA), TaqMan^®^ Universal Master Mix II, with UNG (Applied Biosystems, Warrington, UK). The PCR cycling conditions were 2 min at 50 °C, followed by 10 min at 95 °C, and then 40 cycles of 15 s at 95 °C, 1 min at 60 °C. The reaction mixtures contained 2.5 μL of sample cDNA, 1 μL gene expression assay, and 10 μL TaqMan^®^ Universal Master Mix II, with UNG, and 6.5 μL distilled water. According to the minimum information for publication of quantitative real-time PCR experiments guidelines, β-actin was used as a reference housekeeping gene because of its high stability across various porcine tissues [30,31]. We used DNA plasmids designed by GeneArt Strings^TM^ as standards for qPCR. Plasmids were designed in the 100 bp before and after from the center of the assay location (Total 200 bp). Sequences of the DNA plasmids used are shown in Supplementary Table S1.

### 2.3. Immunohistochemical Analysis

Fresh ileal PPs (*n* = 3) were obtained as described before, washed with phosphate-buffered saline (PBS), cut into small pieces (5 × 10 mm), and fixed in Zamboni’s fixative (Wako, Tokyo, Japan) for 16 h at 4 °C. The fixed tissues were washed for 24 h with 1% gum arabic in 0.1 M phosphate buffer containing 8% sucrose and for an additional 24 h with the same solution containing 16% sucrose. Samples were immersed in TISSUE TEK O.C.T. compound (Sakura Finetechnical, Tokyo, Japan) and quickly frozen in a dry ice/acetone bath. Cryostat sections (10 μm) were prepared from the frozen tissues. The sections were incubated with blocking one histo (Nacalai Tespue Inc., Kyoto, Japan) to block non-specific binding sites. After removal of the blocking solution, sections were incubated for 16 h at 4 °C in a humidified chamber with 1:1000 anti-porcine PGLYRP-3 polyclonal antibody (#COP-080060, Cosmo Bio, Tokyo, Japan) or 1:1000 anti-porcine PGLYRP-4 polyclonal antibody (#COP-080061, Cosmo Bio). After washing with PBS, sections were incubated for 60 min with 1:1000 Alexa 488-conjugated goat anti-rabbit IgG F(ab’)2 (ThemoFisher Scientific, Yokohama, Japan). Double immunostaining for pan-cytokeratin, and either PGLYRP-3 or PGLYRP-4, was also performed using 1:2000 anti-pan cytokeratin monoclonal antibody (clone C-11; Sigma-Aldrich, St. Louis, MO, USA) followed by 1:1000 Alexa 546-conjugated goat anti-mouse IgG F(ab’)2 (ThemoFisher Scientific, Yokohama, Japan). Then, samples were washed three times with PBS and stained with DAPI (Dojindo Laboratories, Kumamoto, Japan) to detect nuclei. Finally, the tissue sections were washed three times with PBS, mounted in ProLong Gold (ThemoFisher Scientific, Yokohama, Japan), and observed under an FSX100 microscope (Olympus, Tokyo, Japan). Control experiments were performed by omitting primary antibodies.

### 2.4. Flow Cytometer-Based Analysis of PGLYRPs Expression in Porcine APCs from Ileal Peyer´s Patches

Expression levels of PGLYRPs in APCs from ileal PPs of adult swine were determined by flow cytometry. APCs were prepared according to our previous studies with some modifications [28,29]. Briefly, PPs were cut into fragments and then smoothly pressed through a nylon mesh, and washed with complete RPMI medium supplemented with 10% FCS. A hypotonic solution (0.2% NaCl, Sigma, Tokyo, Japan) was used to eliminate residual red cells and, a rescue was performed with an equal volume of a hypertonic solution (1.5% NaCl). This mononuclear cell suspension contains a mixed population of T (CD4^+^ and CD8^+^), B (CD21^+^), and APCs (CD4^-^CD8^-^MHC-II^+^) [29]. For the detection of cell surface and intracellular PGLYRPs in different populations APCs (CD172a^high^CD11R1^+^, CD172a^low^CD11R1^+^, or CD172a^low^CD11R1^low^ cells) the following primary antibodies were used: anti-porcine CD172a-PE SWC3 IgG1 (Southern Biotech, Tokyo, Japan), and anti-porcine CD11R1-unlabeled IgG1 (AbD Serotec, Boston, USA). Unlabeled monoclonal antibodies were detected by the following secondary antibodies: anti-mouse IgG1-PerCP/Cy5.5 (Bio Legend, San Diego, CA, USA) and anti-rabbit IgG Alexa Fluor 488. Isotype controls were obtained by incubating cells with isotype mouse IgG1-PE or IgG1-PerCP antibodies (eBioscience, San Diego, CA, USA). The expression of PGLYRP-1 and -2 in APCs was analyzed by using antisera originally obtained from immunized rabbits. The expression of PGLYRP-3 and -4 were analyzed by using the polyclonal antibodies mentioned above. Cells were collected, washed twice with washing buffer (2% FCS, 0.01% NaN_3_/PBS, Sigma, Tokyo, Japan) and live cell counts were adjusted to 1 × 10^6^ cells/tube. Immune cells were resuspended and labeled with primary and secondary antibodies for detection of surface expression of CD172a, CD11R1, and PGLYRP-1, -2, -3 or -4. In another set of experiments, cells were permeabilized with the BD cytofix-cytoperm kit (BD Biosciences, San Jose, CA, USA) according to the manufacturer’s instructions and then labeled with primary anti-mouse PGLYRPs and secondary antibodies for detection of intracellular PGLYRPs. Analysis of the stained cells was performed using BD Accuri™ C6 Flow Cytometer (BD, Franklin Lakes, NJ, USA) equipped with C6 software. FlowJo software (Tree star, Ashland, OR, USA) was used for the data analysis.

### 2.5. Analysis of PGLYRP Expressions in APCs from Peripheral Blood 

Porcine monocyte-derived dendritic cells (MoDCs) were generated as previously described [32]. Briefly, porcine monocytes were obtained from freshly collected porcine peripheral blood by a density gradient centrifugal method (1800 rpm, 20 min, 20 °C) with Lympholyte-mammal (Cedarlane, Hornby, ON, Canada). Cells were suspended in RPMI and plated (1 × 10^7^ cells/mL per well) into 12-well plates (Corning, Brumath, France) in RPMI-1640 supplemented with 2% FCS, 1% Streptomycin/Penicillin, and incubated two hours. Non-adherent cells were removed and remaining cells were incubated with RPMI supplemented with 10 μL/well of porcine GM-CSF (20 ng/mL) and 10 μL/well of porcine IL-4 (20 ng/mL). Fresh medium was renewed every 2 days. After 5 days of culture, cells were incubated with RPMI medium supplemented with GM-CSF, IL-4, and 5 μL/well of LPS (200 μg/mL) in order to induce differentiation of MoDC. The qRT-PCR was performed to quantify the expression of PGLYRPs mRNAs in porcine MoDCs. qRT-PCR was carried out with using Platinum SYBR Green qPCR SuperMix UDG with ROX (Invitrogen, Carlsbad, CA, USA). The primers used in this study are listed in Supplementary Table S2. 

### 2.6. Analysis of PGLYRPs Expression in PIE Cells

The PIE cell line, originally derived from intestinal epithelia from an unsuckled neonatal swine [33,34], was maintained in DMEM (Invitrogen Corporation, Carlsbad, CA, USA) supplemented with 10% FCS, 100 U/mL penicillin, and 100 μg/mL streptomycin at 37 °C in an atmosphere of 5% CO_2_. PIE cells were passaged by treatment with a sucrose/EDTA buffer for 4 min, detached using 0.04% trypsin in PBS, and then plated at a density of 3 × 10^4^ cells/well in a type I collagen-coated (SUMILON, Tokyo, Japan) at 37 °C in an atmosphere of 5% CO_2_. PIE cells were cultured in DMEM supplemented with 10% FCS and passaged every 3 or 4 days. In the present study, PIE cells between the 20th and 35th passages were used in experiments. In order to quantify the expression of PGLYRPs mRNA and protein, qRT-PCR and immunohistochemistry analysis were performed in PIE cells according to methods described earlier. 

### 2.7. Paraimmunobiotic Bifidobacteria

Immunobiotic bifidobacteria strains were provided by Morinaga Milk Industry Co. Ltd. (Zama, Japan). *B longum* subsp. *longum* BB536 and *B. breve* M-16V were grown in Man-Rogosa-Sharpe (MRS) broth and agar (Difco, Detroit, MI, USA) supplemented with 0.05% (*w*/*v*) cysteine (Sigma, Tokyo, Japan) and incubated at 37 °C for 16 h under anaerobic conditions (AnaeroGen; Oxoid, Basingstoke, UK). Cultures were then centrifuged at 19,000× *g* for 10 min, and bifidobacteria were washed with PBS and resuspended in DMEM at the appropriate concentrations [35]. The bifidobacteria were heat-killed at 65 °C for 30 min to obtain the paraimmunobiotics and stored at −80 °C until further use. 

### 2.8. Modulation of PGLYRPs Expression in MoDCs and PIE cells by Paraimmunobiotic Bifidobacteria and/or PRRs Ligands

MoDCs were seeded at 1 × 10^7^ cells/12-well type I collagen-coated plates (Iwaki, Tokyo, Japan) and cultured for 5 days and stimulated with TLR2, TLR4, NOD1, or NOD2 ligands for 12 h. MoDCs (1 × 10^7^ cells/12 well) were also stimulated with the two paraimmunobiotic bifidobacteria individually (5 × 10^7^ CFU/mL) for 6h. PIE cells were seeded at 3 × 10^4^ cells/12-well type I collagen-coated plates (Iwaki, Tokyo, Japan), cultured for 5 days and then stimulated with TLR2, TLR3, TLR4, TLR5, TLR7, TLR8, TLR9, NOD1, or NOD2 ligands for 12 h (Supplementary Table S3). PIE cells were stimulated with the two paraimmunobiotic bifidobacteria individually (5 × 10^7^ CFU/mL) for 6 h. In another set of experiments, PIE cells were seeded at 3 × 10^4^ cells/12-well plate on type I collagen-coated plates (Iwaki), cultured for 3 days and treated with paraimmunobiotic bifidobacteria (5 × 10^7^ cells/mL) for 48 h. Then, each well was washed vigorously with medium at least three times to eliminate bacteria, and cells were stimulated with different PRRs ligands (TLR2, TLR4, NOD2) for 12 h. The mRNA expression of PGLYRPs was evaluated by qRT-PCR.

### 2.9. PRRs Knockdown in PIE Cells

For RNA interference, PIE cells were seeded (3 × 10^4^ cells/well) in 12-well type I collagen-coated plates (Iwaki, Tokyo, Japan) and cultured for 5 days. Then cells were transfected with specific short interfering RNAs (siRNAs) for TLR2 (6.25 pmol), TLR4 (6.25 pmol), NOD1 (12.5 pmol), or NOD2 (12.5 pmol) using lipofectamine RNAiMAX (Invitrogen, Carlsbad, CA, USA) for 12 h. Primers used for TLR2, TLR4, NOD1, and NOD2 gene knockdown in PIE cells are listed in Supplementary Table S4. Stealth RNAi^TM^ siRNA Negative Control Med GC Duplex #3 (#12935-113, Invitrogen, Carlsbad, CA, USA) was used as control. qRT-PCR was performed to examine the inhibition of PRRs expression in knockdown cells. In a second set of experiments, PIE cells were seeded at 3 × 10^4^ cells/12-well type I collagen-coated plates (Iwaki, Tokyo, Japan) and cultured for 5 days. After changing the medium, cells were transfected with siRNAs for 12 h. Then, the PRR knockdown cells were stimulated with paraimmunobiotic bifidobacteria for 6 h to evaluate the regulation of PGLYRP expressions.

### 2.10. Analysis of Intracellular Ca^2+^ flux in PRRs Knockdown PIE Cells

Evaluation of intracellular calcium flux by PRRs ligands in TLR2-, TLR4-, NOD1-, and NOD2-knockdown PIE cells were performed according to the method of Murofushi et al. [36], with some modifications. Briefly, the knockdown cells (1 × 10^5^ cells/100 μL medium) were plated on 96-well Cell Culture Clear Bottom Black Plates (Corning Inc., New York, NY, USA) at 37 °C for 24 h. After the careful elimination of the medium by aspiration, 100 μL lording buffer of Calcium Kit-Fluo 4 (Dojindo Laboratories, Tokyo, Japan) was added and incubated at 37 °C for 1 h. The medium was then replaced by 100 μL of the recording medium. The dye-stained cells were washed with PBS and stimulated with 10 μL (1 mg/mL) of LTA, LPS, DAP, or MDP by the dropping system in a fluorescence spectrophotometer (ARVO^TM^ X3, PerkinElmer Inc., Billerica, MA, USA). The fluorescence intensity was recorded up to 140 s after stimulation. 

### 2.11. PGLYRPs Knockdown in PIE Cells

For the generation of PGLYRP-1-, PGLYRP-2-, PGLYRP-3-, and PGLYRP-4-knockdown cells, PIE cells were seeded at 3 × 10^4^ cells/12-well type I collagen-coated plates, and cultured as described earlier in section “PRRs knockdown in PIE cells”. Cells were transfected with specific siRNAs for PGLYRP-1, -2, -3 or -4 (26.6 pmol). Primer sets used for PGLYRPs gene knockdown in PIE cells are listed in Supplementary Table S5. Stealth RNAi^TM^ siRNA Negative Control Med GC Duplex #3 (#12935-113, Invitrogen, Carlsbad, CA, USA) was used as control. The qRT-PCR was performed by TaqMan probe method with corresponding primers for PGLYRP-1, -2, -3, and -4 to examine the inhibition of PGLYRPs expressions in knockdown cells. PGLYRP knockdown PIE cells were stimulated with paraimmunobiotic bifidobacteria for 6 h and qRT-PCR was used to evaluate the expression of TLR2, TLR4, NOD1, and NOD2. 

### 2.12. Statistical Analysis 

The results are shown as means ± standard deviation obtained from three independent experiments (*n* = 6). The qRT-PCR raw data were log-transformed followed by normality check by Kolmogorov-Smirnov test and convergence by clubs rejection test. Data were corrected for the non-stimulated control to become 1.0. One-way ANOVA was performed in GraphPad prism v5.1 followed by calculating the Fisher’s least significant difference for multiple mean comparisons were defined as significant at *p* < 0.05 or *p* < 0.01. 

## 3. Results

### 3.1. Expression Patterns of PGLYRPs in Porcine Tissues

In a previous study, we cloned and characterized porcine PGLYRP-3 and PGLYPR-4 [15]. Our qRT-PCR-based expression analysis revealed that porcine PGLYRP-3 and -4 are strongly expressed in the digestive tract of newborn and adult pigs. We also constructed transfected cell lines in order to explore the subcellular distribution of porcine PGLYRP-1 to -4. Results indicated that porcine PGLYRP-1, -3, and -4 are secreted from cells while PGLYRP-2 and PGLYRP-3 are strongly and weakly expressed on the cell surface, respectively [15]. However, quantitative comparisons of tissue-specific expression of the four porcine PGLYRP genes and the confirmation of their expression at the protein level have remained elusive. Therefore, in the present study, we first aimed to evaluate the expression of the four porcine PGLYRPs in Peyer´s patches (PP) by using qRT-PCR and immunofluorescence.

**Figure 1:**
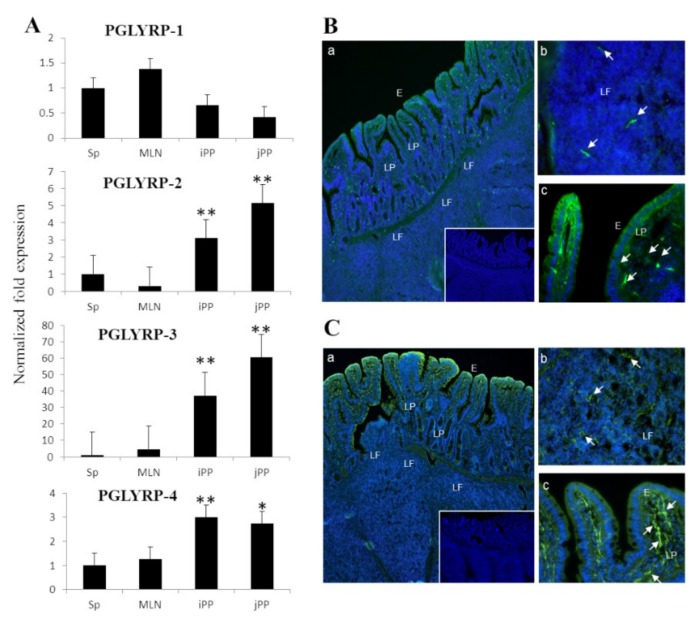
Expression analysis of peptidoglycan recognition proteins (PGLYRPs) in porcine tissues. PGLYRPs expression in the porcine spleen (Sp), mesenteric lymphoid nodes (MLN), and Peyer´s patches from the ileum (iPP) or jejunum (jPP) analyzed by real-time quantitative PCR (**A**). The PGLYRP mRNA expression level was normalized by using porcine β-actin mRNA and the relative index was determined by comparison to the PGLYRP mRNA level in the spleen (1.00). Immunohistochemical analysis of PGLYRP-3 (**B**) and PGLYRP-4 (**C**) expressions are displayed for ileal Peyer’s patches. Images represent the expression of PGLYRPs in the whole iPP (a), in the lymphoid follicles of iPP (b), and in the lymphoid follicle epithelium of iPP (c). The arrow indicates the presence of dendritic-like cells with a strong expression of PGLYRP-3 or PGLYRP-4. E: epithelium, LP: lamina propria, LF: lymphoid follicle. Values represent the means and error bars indicate the standard deviation. The results are shown as means ± standard deviation obtained from three independent experiments (*n* = 6). * *p* < 0.05, ** *p* < 0.01 against spleen.

The expression of porcine PGLYRPs in the spleen was set as reference (as 1.00) and compared with those expressed in ileal PP, jejunal PP, and mesenteric lymph nodes (MLN) (Figure 1A). PGLYRP-2, -3, and -4 were detected in both ileal and jejunal PPs while the expression of PGLYRP-1 in those tissues was lower than in the spleen. PGLYRP-1, -3, and -4 were detected in MLN while PGLYRP-2 was weakly expressed in this tissue (Figure 1A).

### 3.2. Immunohistochemical Localization of PGLYRP Proteins in the Porcine Tissues

Immunofluorescence studies were performed to evaluate the expression of PGLYRP-3 and -4 proteins in porcine ileal PPs (Figure 1B,C). Both PGLYRPs (green fluorescence) were expressed in not only the follicle-associated epithelia but also in dendritic-like cells located beneath the epithelium (Figure 1B,C). 

In addition, PGLYRP-3 or -4 expressing cells were found in the lymphoid follicles. These results would indicate that PGLYRP-3 and -4 are expressed in porcine IECs and APCs. Then, we performed additional experiments in IECs and APCs to demonstrate the expression of PGLYRPs in those cell populations.

### 3.3. Expression Patterns of PGLYRPs in Porcine APCs of Peyer´s Patches

**Figure 2:**
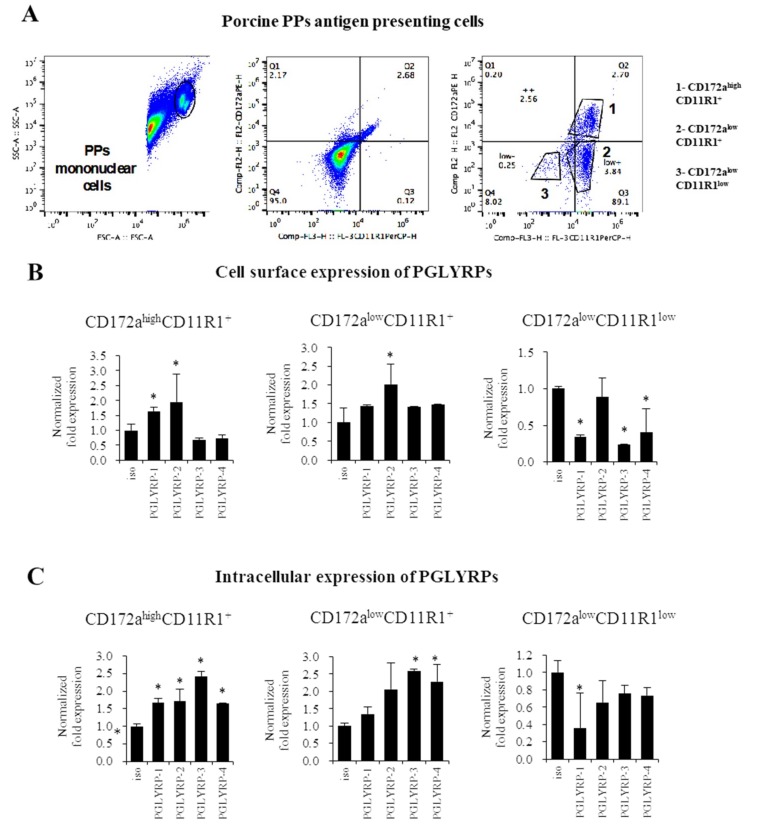
Expression and cellular localization of Peptidoglycan Recognition Proteins (PGLYRPs) in antigen-presenting cells (APCs) from porcine Peyer’s patches (PPs). Flow cytometric analysis of APCs populations from porcine PPs (**A**). APCs were divided into three populations using CD172a and CD11R1 markers and PGLYRP-1, -2, -3, and -4 expressions were evaluated in the surface (**B**) and in the cytoplasm (**C**). The results are shown as means ± standard deviation obtained from three independent experiments (*n* = 6). * *p* < 0.05 against isotype control (iso).

We next aimed to characterize the expression of PGLYRPs in different subsets of APCs isolated from porcine PPs. We used CD172a and CD11R1 markers to define three different populations of MHC-II^+^ APCs [32,37] as CD172a^high^CD11R1^+^, CD172a^low^CD11R1^+^, or CD172a^low^CD11R1^low^ cells (Figure 2A). 

Flow cytometric analysis revealed that PGLYRP-2 is strongly expressed in the cell surface of CD172a^high^CD11R1^+^ and CD172a^low^CD11R1^+^ cells (Figure 2B). The CD172a^high^CD11R1^+^ population also showed expression of surface PGLYRP-1. It was also found that the four PGLYRPs were expressed in the intracellular compartment of CD172a^high^CD11R1^+^ cells and that CD172a^low^CD11R1^+^ cells intracellularly expressed PGLYRP-3 and -4 (Figure 2C). The CD172a^low^CD11R1^low^ cells had no expression of PGLYRPs either on the cell surface (Figure 2B) or in the cell cytoplasm (Figure 2C).

### 3.4. Expression Patterns of PGLYRP in MoDCs and Their Modulation by PRR-ligands and Paraimmunobiotics

**Figure 3:**
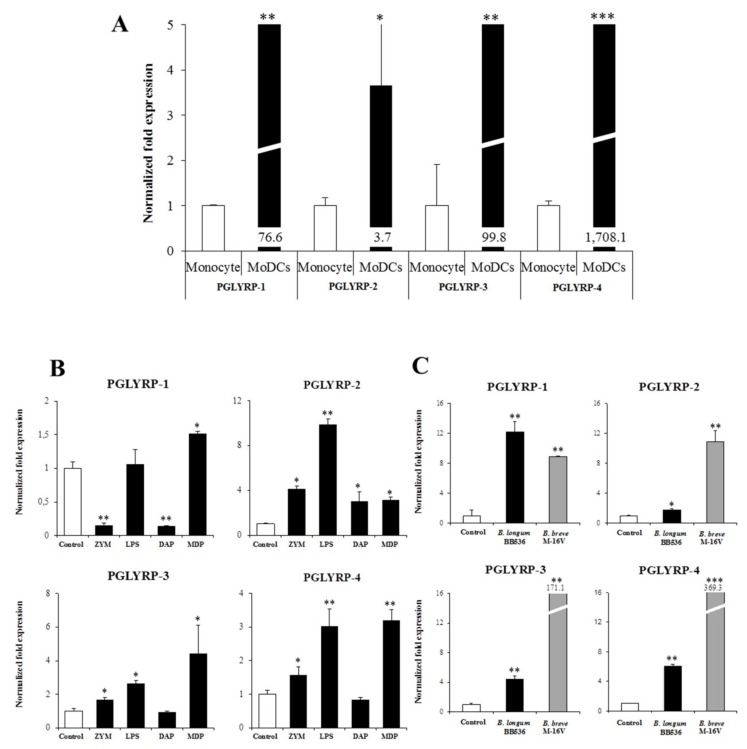
Peptidoglycan recognition proteins (PGLYRPs) expression in porcine blood monocytes and monocyte-derived dendritic cells (MoDCs) and their modulation by pattern recognition receptor (PRR)-ligands and paraimmunobiotic bifidobacteria. Expressions of PGLYRPs in porcine blood monocytes and MoDCs without stimulation (**A**). PGLYRPs expressions in porcine MoDCs stimulated with PRR-ligands: zymosan (ZYM), lipopolysaccharide (LPS), muramyl tripeptide (DAP), and muramyl dipeptide (MDP) (**B**). PGLYRPs expressions in porcine MoDCs stimulated with paraimmunobiotic bifidobacteria: *Bifidobacterium longum* subsp. *longum* BB536 or *B. breve* M-16V (**C**). PGLYRP mRNA expression level was normalized using the porcine β-actin mRNA, and the relative index was determined by comparison to the PGLYRP mRNA level in monocytes or control MoDCs (1.00). Values represent the means ± standard deviation obtained from three independent experiments (*n* = 6). * *p* < 0.05, ** *p* < 0.01, *** *p* < 0.001 against either monocytes (**A**), or control (**B**).

We have previously compared the immune response to microorganisms of porcine APCs originated from PPs and MoDCs; we also observed that immature MoDCs have a similar behavior to that of APCs from mucosal origin [32]. We, therefore, aimed herein to compare the expression of porcine PGLYRPs in blood monocytes and in MoDCs. Results showed that expressions of the four PGLYRPs were significantly higher in MoDCs when compared to blood monocytes, especially for the PGLYRP-4 (Figure 3A). 

We evaluated the effect of different PRR-ligands on the expression of porcine PGLYRPs in MoDCs including zymosan, LPS, DAP-containing muramyl tripeptide (DAP) and muramyl dipeptide (MDP) which are ligands for TLR2, TLR4, NOD1, and NOD2, respectively (Figure 3B). Activation of TLR2 in porcine MoDCs significantly increased the expression of PGLYRP-2, -3 and -4 while it decreased PGLYRP-1. TLR4 activation increased PGLYRP-2, -3 and -4 expressions (Figure 3B). In addition, stimulation of NOD1 decreased PGLYRP-1 and increased PGLYRP-2 expression while NOD2 activation significantly augmented the expression of the four PGLYRPs in porcine MoDCs (Figure 3B).

We also evaluated the effect of two paraimmunobiotic bifidobacteria on the modulation of PGLYRPs expression in porcine MoDCs (Figure 3C). Stimulation of MoDCs with *Bifidobacterium longum* subsp. *longum* BB536 or *Bifidobacterium breve* M-16V significantly increased the expression of the four PGLYRPs. The most remarkable changes were observed for PGLYRP-3 and -4 expressions in the porcine MoDCs treated with the M-16V strain.

### 3.5. Expression Patterns of PGLYRPs in PIE Cells and Their Modulation by PRR-ligands

**Figure 4:**
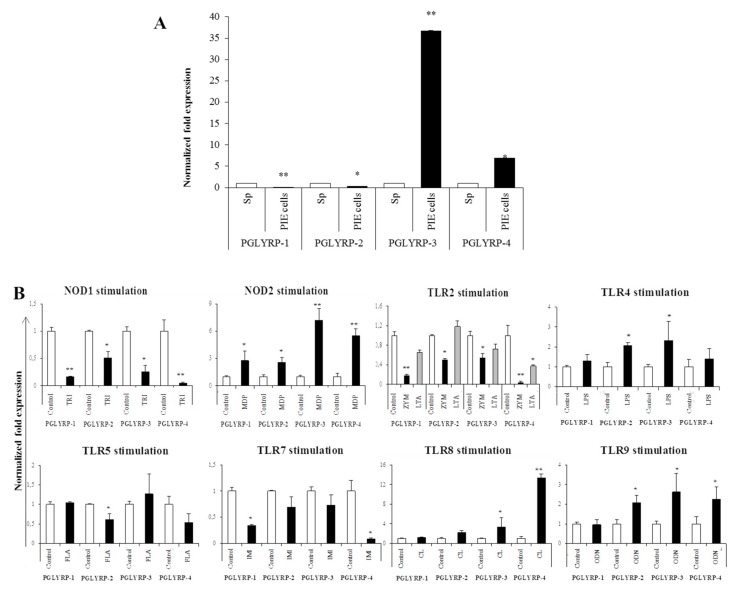
Peptidoglycan recognition proteins (PGLYRPs) expression in porcine intestinal epithelial (PIE) cells and their modulation by pattern recognition receptor (PRR)-ligands. Expression of PGLYRP-1, -2, -3, and -4 in the porcine spleen and in PIE cells without stimulation (**A**). Modulation of PGLYRP-1, -2, -3, and -4 expressions in PIE cells after stimulation with eight different PRR-ligands (**B**). Zymosan (ZYM) and lipoteichoic acid (LTA) were used as TLR2-ligand. Lipopolysaccharide (LPS), flagellin (FLA), imiquimod (IMI), CL075 (CL), ODN2006 (ODN), muramyl tripeptide (DAP), and muramyl dipeptide were used as ligands for TLR4, TLR5, TLR7, TLR8, TLR9, NOD1, and NOD2, respectively. Expressions of PGLYRP mRNA level was normalized using the porcine β-actin mRNA, and the relative index was determined by comparison to the PGLYRP mRNA level in non-stimulated PIE cells (1.00). Values represent the means ± standard deviation obtained from three independent experiments (*n* = 6). * *p* < 0.05 and ** *p* < 0.01 against control cells.

We characterized the expression patterns of PGLYRPs in PIE cells, which are cell lines that were originally established by our group for the selection of immunobiotic candidates as well for the evaluation of immune responses in porcine IECs [29,34,38]. Here, we evaluated the mRNA expression levels of the four PGLYRPs in PIE cells in comparison with healthy porcine spleen tissue. It was found that PGLYRP-3 and -4 showed high expressions compared to PGLYRP-1 and -2 (Figure 4A).

The capacity of different PRR-ligands to modulate the expression of PGLYRPs in PIE cells was also evaluated. TLR2 activation in PIE cells reduced the expression of PGLYRPs. Zymosan stimulation significantly reduced the expression of the four PGLYRPs while LTA only reduced PGLYRP-4 (Figure 4B). NOD1 activation by DAP stimulation reduced the expression of the four PGLYRPs while NOD2 activation by MDP stimulation increased all the PGLYRPs in PIE cells (Figure 4B). Stimulation of PIE cells with LPS increased the expression of PGLYRP-2 and -3. Activation of TLR5 with its ligand flagellin decreased PGLYRP-2 (Figure 4B). TLR7 activation by CL075 decreased the expression of PGLYRP-1 and -4 while TLR8 activation by Imiquimod enhanced the PGLYRP-3 and -4 expressions in PIE cells (Figure 4B). Significant upregulation of PGLYRP-2, -3, and -4 expressions were observed when PIE cells were treated with the TLR9 agonist ODN2006 (Figure 4B).

### 3.6. Modulation of PGLYRPs Expression in PIE Cells by Paraimmunobiotic Bifidobacteria

**Figure 5:**
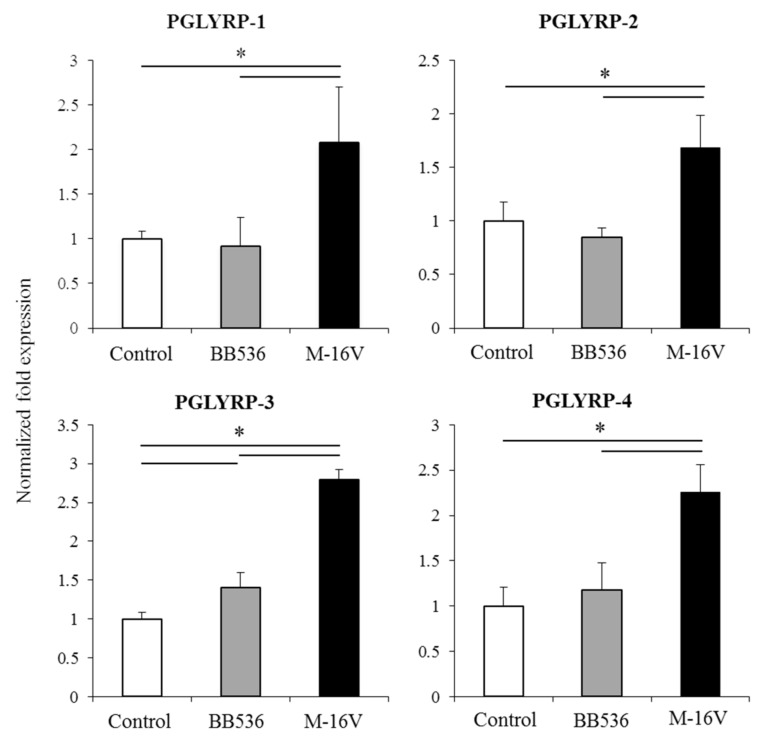
Modulation of the peptidoglycan recognition proteins (PGLYRPs) expression in porcine intestinal epithelial (PIE) cells by paraimmunobiotic bifidobacteria. PIE cells were stimulated with paraimmunobiotic *Bifidobacterium longum* subsp. *longum* BB536 or *B. breve* M-16V. The qRT-PCR-based expression of PGLYRPs mRNA level was normalized using the porcine β-actin mRNA, and the relative index was determined by comparison to the PGLYRPs mRNA level in non-stimulated PIE cells (1.00). The results are shown as means ± standard deviation obtained from three independent experiments (*n* = 6). * *p* < 0.05 against control or against the indicated experimental group.

The effect of the two paraimmunobiotic bifidobacteria strains on the regulation of PGLYRPs expressions in PIE cells was evaluated. The expressions of the four PGLYRPs were increased in PIE cells after their treatment with *B. longum* subsp. *longum* BB536 or *B. breve* M-16V. Of note, *B. breve* M-16V was more efficient than the BB536 strain to enhance the expression of porcine PGLYRPs in PIE cells (Figure 5).

**Figure 6:**
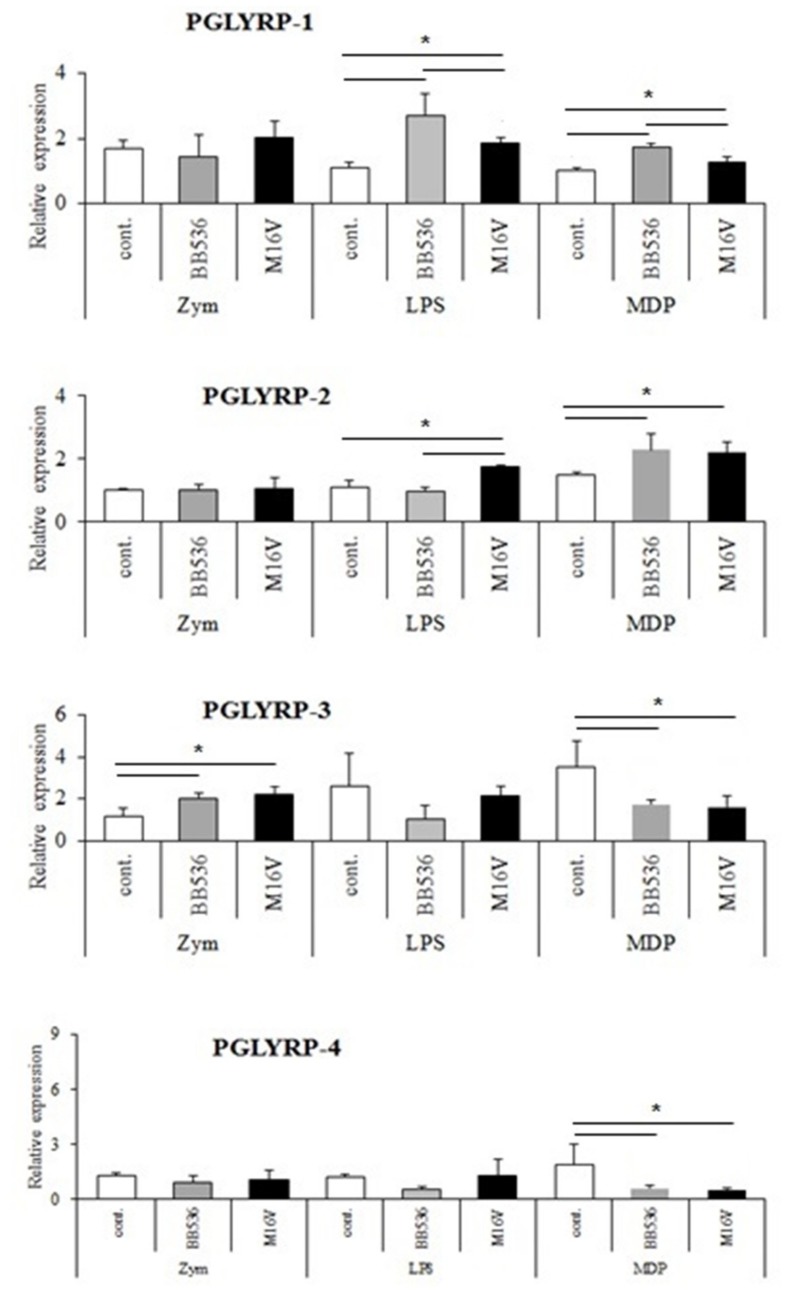
Modulation of peptidoglycan recognition proteins (PGLYRP) expressions by the interactions of pattern recognition receptor (PRR)-ligands and immunobiotics in porcine intestinal epithelial (PIE) cells. The PIE cells were pre-stimulated with *Bifidobacterium longum* subsp. *longum* BB536 or *B. breve* M-16V followed by the stimulation with zymosan (ZYM), lipopolysaccharide (LPS) or Muramyl dipeptide (MDP). PGLYRP mRNA expression level was normalized with the porcine β-actin mRNA, and the normalized fold expression was determined in comparison to the PGLYRP mRNA level of non-stimulated PIE cells. The results are shown as means ± standard deviation obtained from three independent experiments (*n* = 6). * *p* < 0.05 against control or against the indicated experimental group.

We further explored the potential interactions between PRRs ligands and the two bifidobacteria strains in the modulation of PGLYRPs expressions in PIE cells. For this purpose, the epithelial cells were first exposed to BB536 and M-16V strains followed by treatments with zymosan, LPS or MDP. BB536 or M-16V treatments did not change the expressions of PGLYRP-1, -2, or -4 in PIE cells stimulated with zymosan. However, both paraimmunobiotics increased PGLYRP-3 expression PIE cells after zymosan treatment (Figure 6). Bifidobacteria had no effects on the expressions of PGLYRP-3 and -4 in PIE cells after LPS stimulation. M-16V strain increased the expression of PGLYRP-1 and-2 while B536 strain increased only PGLYRP-1 after the stimulation of PIE cells with LPS. Both bifidobacteria increased PGLYRP-1 and -2 and, decreased PGLYRP-3 and -4 expressions n PIE cells after NOD2 stimulation (Figure 6).

### 3.7. Modulation of PGLYRPs Expression by Paraimmunobiotics in PRR-knockdown PIE Cells

**Figure 7:**
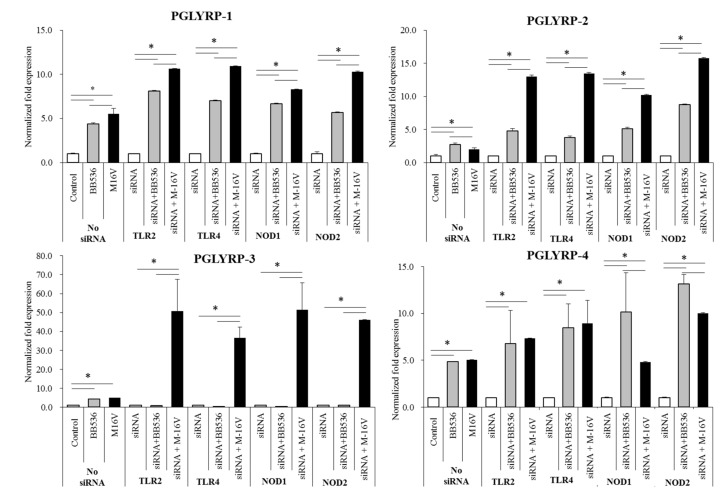
Paraimmunobiotic bifidobacteria-mediated peptidoglycan recognition proteins (PGLYRP) expressions in pattern recognition receptor (PRR)-knockdown porcine intestinal epithelial (PIE) cells. Porcine PGLYRP-1, -2, -3 and -4 expressions in PIE cells transfected with TLR2, TLR4, NOD1, and NOD2 siRNA, and subsequently stimulated with *Bifidobacterium longum* subsp. *longum* BB536, or *B. breve* M-16V. The porcine PGLYRP mRNA level was normalized with the porcine β-actin mRNA, and the normalized fold expression was determined in comparison to the PGLYRP mRNA level of non-stimulated PIE cells. The results are shown as means ± standard deviation obtained from three independent experiments (*n* = 6). * *p* < 0.05 against control or against the indicated experimental group.

In order to evaluate the effect of the interactions between paraimmunobiotic bifidobacteria and PRRs in the modulation of PGLYRPs expressions in PIE cells, we next generated knockdown PIE cells for four PRRs: TLR2, TLR4, NOD1, or NOD2. The RNA interference technology was employed to knockdown each PRR’s expression in PIE cells using specific siRNAs with high inhibition capacity. For each receptor, three different siRNAs were used to transfect PIE cells and the knockdown of PRR’s expression was evaluated by qRT-PCR. In addition, the abolishment of PRR´s function was evaluated by studying the induction levels of intracellular Ca^2+^ fluxes after specific ligand treatment (Supplementary Figure S1A–D). The induction of intracellular Ca^2+^ fluxes was evaluated by recording the fluorescence intensity. Interestingly, the ability of *B. breve* M-16V to increase the expression of PGLYRP-1 and 2 was significantly improved in TLR2-, TLR4-, NOD1-, and NOD2-knockdown cells (Figure 7). The capacity of *B. breve* M-16V to enhance the expression of PGLYRP-4 was significantly increased in TLR4-, and NOD2-knockdown PIE cells compared with normal PIE cells stimulated with the bacterium. The expression of PGLYRP-3 was significantly enhanced by the M-16V treatment in all knocked down PIE cells. An enhancement of PGLYRP-1 expression by *B. longum* subsp. *longum* BB536 stimulation was observed in all PRR-knockdown cells. In addition, the BB536 treatment also results in enhanced PGLYRP-2 expression in TLR2-, and TLR4-knockdown PIE cells; PGLYRP-3 expression in TLR2-, and NOD1-knockdown PIE cells; and PGLYRP-4 expression in TLR4-, NOD1-, and NOD2-knockdown PIE cells (Figure 7).

### 3.8. Modulation of PRRs Expression by Paraimmunobiotics in PGLYRP-knockdown PIE Cells

**Figure 8:**
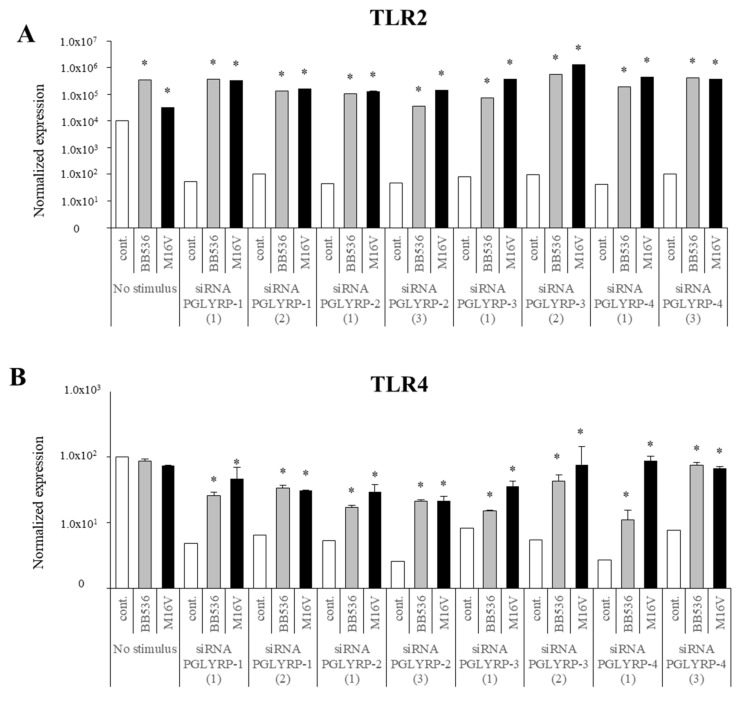
Paraimmunobiotic bifidobacteria-mediated expression of TLR2 and TLR4 in peptidoglycan recognition proteins (PGLYRP)-knockdown porcine intestinal epithelial (PIE) cells. Porcine TLR2 (**A**) and TLR4 (**B**) expressions in PIE cells transfected with PGLYRP siRNAs, and stimulated with *Bifidobacterium longum* subsp. *longum* BB536, or *B. breve* M-16V. Each mRNA level was normalized with the porcine β-actin mRNA and the normalized fold expression was determined in comparison to PIE cells with transfected negative control siRNA. The results are shown as means ± standard deviation obtained from three independent experiments (*n* = 6). * *p* < 0.05 against control.

**Figure 9:**
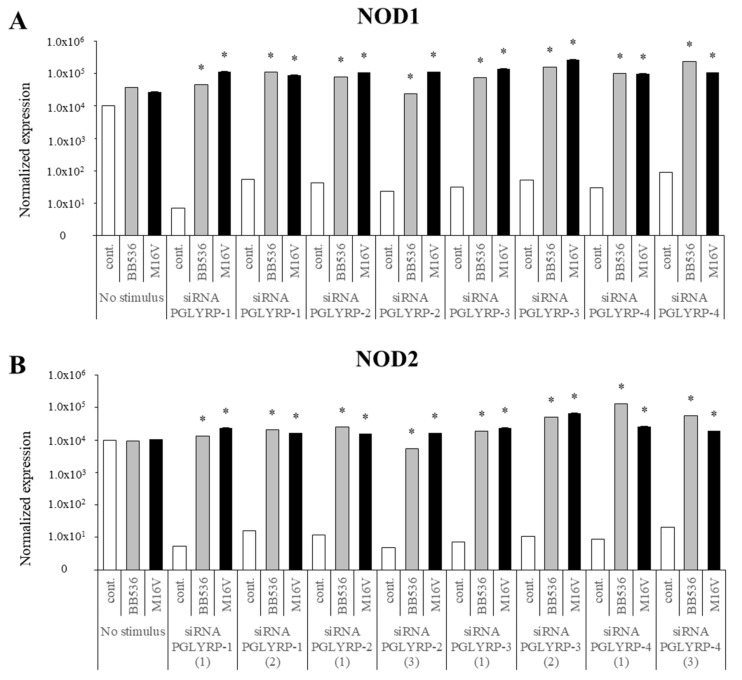
Paraimmunobiotic bifidobacteria-mediated expression of NOD1 and NOD2 in the peptidoglycan recognition proteins (PGLYRP)-knockdown porcine intestinal epithelial (PIE) cells. Porcine NOD1 (**A**) and NOD2 (**B**) expressions in PIE cells transfected with PGLYRP siRNAs and stimulated with *Bifidobacterium longum* subsp. *longum* BB536, or *B. breve* M-16V. Each mRNA level was normalized by the porcine β-actin mRNA and the normalized fold expression was determined in comparison to PIE cells with transfected negative control siRNA. The results are shown as means ± standard deviation obtained from three independent experiments (*n* = 6). * *p* < 0.05 against control.

Finally, we generated knockdown PIE cells for the four PGLYRPs and evaluated the effect of paraimmunobiotic bifidobacteria in PRRs expressions. For each PGLYRP, three different siRNAs were used to transfect PIE cells and the knockdown efficiency was evaluated by qRT-PCR-based expression of PGLYRP genes in the transfected cells (Supplementary Figure S2A–D). Then, two successful knocked down cell populations for each gene were used to evaluate the paraimmunobiotic bifidobacteria mediated immunomodulation. We observed that the expression of TLR2, TLR4, NOD1, and NOD2 was significantly reduced in the four PGLYPR-knockdown cell populations as compared to that of non-transfected PIE cells (Figure 8 and Figure 9). Interestingly, *B. breve* M-16V and *B. longum* subsp. *longum* BB536 treatments increased TLR2, NOD1, and NOD2 expressions in all PGLYPR knocked down PIE cells (Figure 8 and Figure 9). In addition, both paraimmunobiotic bifidobacteria significantly enhanced the expression of TLR4 in PGLYRP-1-, PGLYRP-2-, and PGLYRP-4-knockdown PIE cells. Although both BB536 and M-16V strains increased TLR4 expression in PGLYRP-3-knockdown PIE cells, *B. breve* M-16V was more efficient to induce this effect (Figure 8).

## 4. Discussion

Pigs are livestock animals of significant economic importance. In addition, because of the anatomical and physiological similarities between pigs and humans, these animals have attracted attention as valuable models for immunological research [39,40,41]. In this regard, porcine in vivo and in vitro studies have provided insights into molecular mechanisms involved in the interaction of the mucosal immune system with pathogenic and beneficial microbes [33,37,42]. Several innate immune receptors possessing the ability to regulate immune responses have been identified in human and murine intestinal mucosa [5]; PGLYRP family is one of the major classes of them. Earlier studies in pigs showed that PGLYRP-2 is expressed constitutively in several tissues including liver, spleen, and intestine [29]. Likewise, our previous report also revealed that PGLYRP-3 and -4 are highly expressed in the digestive tract of newborn and adult pigs [15]. In the present study, we assessed the expression profiles of four PGLYRPs at gene and protein levels simultaneously in porcine PP. Results demonstrated that PGLYRP-1, PGLYRP-2, PGLYRP-3, and PGLYRP-4 are expressed in this porcine tissue. In addition, we observed that PGLYRP-3 and -4 proteins were highly expressed in IECs, which is consistent with the findings of previous reports conducted in humans and mice [3,8,13]. 

To the best of our knowledge, we are first in demonstrating that the four PGLYRPs are expressed in the porcine APCs of both mucosal and non-mucosal origin. PGLYRPs have known to be involved in phagocytic activity, pathogen elimination, increased monocyte activation and pro-inflammatory cytokine secretion [11]. The expression of PGLYRP-3 and PGLYRP-4 proteins have been detected in murine macrophages of the spleen as well as in the macrophage-like cell line RAW264.7 [8]. Expression of PGLYRPs have also been detected in human blood phagocytes [11]. Flow cytometric studies showed that PGLYRP-1, PGLYRP-3, or PGLYRP-4 antibodies bound preferentially to monocytes rather than to lymphocytes [11]. In addition, it was shown that the inflammatory response triggered by PGN in human monocytes was increased in the presence of PGLYRP-PGN complexes compared with PGN alone. Moreover, the presence of PGLYRP-PGN complexes diminished the anti-inflammatory responses [11]. These findings support our speculation that PGLYRPs would have an important role in the interaction of mucosal and non-mucosal APCs with microorganisms and their ligands and, in the generation of immune responses in pigs.

The expressions of PGLYRPs in porcine APCs and IECs were found to be modulated by the stimulation of different PRR-ligands. The most notable effect was observed after NOD2 activation that induced an upregulation of the four PGLYRPs in porcine MoDCs and PIE cells. In addition, TLR4 activation significantly increased the expression of PGLYRP-2 and -3 in MoDCs and PIE cells. Those findings are in agreement with the findings of Uehara et al. [43], who observed that PGLYRP expressions in human oral epithelial cells upon stimulation with different chemically synthesized PAMPs were markedly upregulated via activation of TLR4 or NOD2. The work also reported that TLR2 and NOD1 activation increased the expression of PGLYRPs [43]. In contrast with those results, we observed here that stimulation of PIE cells with zymosan or NOD1 ligands significantly decreased the expression of PGLYRP-1, -2, -3, and -4. These results imply that TLR2 and NOD1 would have negative regulatory roles in the expression of PGLYRPs in porcine IECs. This is in line with the report of Jang et al. [41] who showed that PGLYRP negatively regulates NOD-mediated cytokine production in rainbow trout live cell. However, this PRR-mediated regulatory role could be cell-type specific, since TLR2 or NOD1 stimulation has significantly increased the expression of PGLYRP-2 in porcine MoDCs. Interestingly, the stimulation of PIE cells with zymosan significantly reduced the expression of the four porcine PGLYRPs, while stimulation with LTA did not induce modifications in PGLYRPs. PGLYRPs bind to bacterial PGN but may not necessarily respond to exogenous TLR2 ligands, since a recent report revealed that neither Lys-and DAP-type PGN stimulate mouse or human innate immune cells via TLR2 [40]. Zymosan is a crude extract of *Saccharomyces cerevisiae* widely used as TLR2 ligand, while this PRR did not sense the highly purified PGN from *Bacillus anthracis* and *Staphylococcus aureus*, though the live bacteria of both species express abundant of TLR2 ligands [40]. 

It was also demonstrated here that paraimmunobiotic bifidobacteria were able to increase the expression of PGLYRPs in both MoDCs and PIE cells. The most remarkable effects of paraimmunobiotic treatments were observed for PGLYRP-3 and -4. It is known that PGLYRP-1, -3 and -4 are bactericidal for pathogenic Gram-positive bacteria, and bacteriostatic for most Gram-positive and Gram-negative commensal bacteria [13]. On the other hand, PGLYRP-2 is an *N*-acetylmuramyl-l-alanine amidase and is bacteriostatic in nature. Pathogenic Gram-positive bacteria, such as *Listeria monocytogenes* and *Staphylococcus aureus* are highly sensitive to the bactericidal effects of PGLYRP-3 and PGLYRP-4 while the Gram-positive commensal bacterium *Enterococcus faecalis* showed much less sensitivity to PGLYRP-mediated killing [13]. Though pathogenic bacteria (*Staphylococcus aureus*) led to a rapid onset of PGLYRP-3 expression that decreased afterward, while the immunobiotic bacteria *Lactobacillus rhamnosus* GG resulted in a slow but sustained expression of PGLYRP-3 [18]. On the other hand, the immunobiotic strains *Lactobacillus jensenii* TL2937 and *Lactobacillus rhamnosus* CRL1505 were able significantly improve PGLYRP-2 expression in PIE cells [38,44]. It is therefore conceivable that commensal gut microbiota would be able to improve the expression of PGLYRPs in the intestinal mucosa and increase the bactericidal activities against pathogens. The enhanced activities of PGLYRPs would not significantly affect commensals since they develop resistance to the bactericidal mechanisms mediated by PGLYRP-1, PGLYRP-3, and PGLYRP-4. It should be also considered that bifidobacteria used in this work were not viable, and therefore, they would not be affected by the activities of PGLYRPs. To investigate in vivo the effect of paraimmunobiotic bifidobacteria on porcine intestinal PGLYRPs expression and to evaluate whether these changes influence the resistance to intestinal pathogens are interesting topics for future near research.

The expression of TLR2, TLR4, NOD1, and NOD2 in PGLYRP-knockdown PIE cells were reduced but PGLYRPs expressions in PRR-knockdown PIE cells remained stable when un-stimulated knockdown cells were compared to their respective non-transfectant control cells. These results indicate that PGLYRPs expressions would be required for the activation and functioning of TLR2/4 and NOD1/2, while PGLYRPs activation would be independent of TLR2/4 and NOD1/2 expressions. Surprisingly, stimulation with paraimmunobiotic bifidobacteria, particularly the M-16V strain, resulted in a significant upregulation of PGLYRP-1, -2, and -3 expressions in the PRR-knockdown PIE cells. Mice knockdown for any one of the four PGLYRP genes has been reported to be more sensitive to dextran sulfate sodium (DSS)-induced colitis and dysbiosis of gut microbiota [19,20]. The main pathologic changes by PGLYRPs-deficiency include the loss of normal colon tissue architecture due to loss of epithelium, hyperplasia of the lamina propria, and resulting inflammation [19]. Interestingly, it was reported that PGLYRP-2^−/−^ and PGLYRP-3^−/−^ mice were highly sensitive to DSS-induced colitis than PGLYRP-1^−/−^ and PGLYRP-4^−/−^ mice, which had intermediate or low sensitivity, respectively [19]. Notably, PGLYRP-3^−/−^ mice had the most significant changes in their gut microbiota whereas PGLYRP-1^−/−^ and PGLYRP-4^−/−^ mice were less sensitive to colitis and had fewer alterations in the gut microbiota [19]. Together these findings strongly suggest the potential of paraimmunobiotic *B. breve* M-16V for modulating PGLYRP expression in IECs and minimizing the pathogenesis of intestinal inflammatory diseases through beneficial modification of intestinal innate immunity via PGLYRPs. In line with this hypothesis, it was demonstrated that administration of viable *B. breve* M-16V reduced the pathological scores of necrotizing enterocolitis-related inflammation in a preterm rat model [28] and ameliorated DSS-induced inflammation in weanling rats [45]. The in vivo demonstration that the non-viable bacterium retains these immunomodulatory effects would be of fundamental importance considering that it could allow its application in populations where the administration of a living microorganism represents a potential danger, such as immunocompromised hosts.

Our results suggest that NOD2 would be involved, at least partially, in the regulatory effect of bifidobacteria in PGLYRP expressions since the NOD2 agonist was the only PRR ligand able to increase the expression of the four PGLYRPs in a similar trend as bifidobacteria. Both PGLYRP-3 and NOD2 recognize the bacterial PGN and play a synergistic role in maintaining intestinal microbiota and inflammation as PGLYRP-3^−/−^ NOD2^−/−^ double-knockout mice were more sensitive to DSS-colitis than PGLYRP-3^−/−^ or NOD2^−/−^ single knockout animals [46]. NOD2 and PGLYRP-2 showed a synergistic effect in the development of local inflammation in an arthritis mice model since PGLYRP2^−/−^ NOD2^−/−^ mice were resistant to PGN or MDP induced arthritis [47]. Another study reported PGLYRP2^−/−^ and NOD2^−/−^ double knockdown mice had an increased susceptibility to *Salmonella* infection as compared with single PGLYRP2^−/−^ knockdown mice [46], which indicate that PGLYRP2 plays a protective role in the control of *S. enterica* serovar Typhimurium infection in vivo through NOD2-dependent mechanism. It is, therefore, possible to think that the effect of paraimmunobiotic bifidobacteria would be the result of a combination of the activation of at least two PRRs (TLR2 and NOD2) and that the expression of the PGLYRPs would be the consequence of the interactions between these two signaling pathways. Identification of the PRRs and signaling pathways involved in PGLYRPs regulation by paraimmunobiotics in porcine APCs and IECs is an interesting topic for future investigations. In addition, to find out the functional molecules present in paraimmunobiotic bifidobacteria that are able to trigger TLR2 and NOD2 activation is of interest, therefore, to explore their roles in the regulation of PGLYRPs expressions in the porcine IECs and APCs. 

Multiple experimental studies demonstrated an increased formation of superoxide, hydrogen peroxide, hypochlorous acid, and peroxynitrite in colonic mucosa in animal models of IBD [48]. During the early stage of the disease process and even before the genesis of IBD, IECs were shown to produce elevated levels of reactive oxygen and nitrogen species through the activation of NOX and iNOS enzymes [48]. Studies in flies have demonstrated that the deletion of PGLYRPs induce the overgrowth of bacteria in the fly gut and promoted intestinal damage, increased proliferation of intestinal stem cells, and dysplasia through the activation of the intestinal NADPH oxidase NOX and the generation of reactive oxygen species [49]. On the other hand, it was reported that the deletion of NOD2 in IECs enhance the internalization of bacteria by epithelia. This uptake of bacteria was dependent on reactive oxygen species and MAP-kinase activity, and the increased viable intracellular bacteria in NOD2^−/−^ cells reflected a reduced ability to recognize and kill bacteria [50]. Then, a significant barrier defect occurs together with PGLYRPs-NOD2 alterations in conjunction with oxidative stress that could contribute to inflammation and development of IBD. To evaluate the potential beneficial effect of paraimmunobiotic bifidobacteria on the oxidative stress in the context of gastrointestinal damage mediated by PRRs-triggered inflammation is also an interesting topic for future research.

Of note, as described for other probiotic or immunobiotic properties, we found that the capacity of paraimmunobiotic bifidobacteria of regulating PGLYRPs expressions in porcine APCs and IECs was strain-dependent. Indeed, our results showed that *B. breve* M-16V was more efficient than *B. longum* subsp. *longum* BB536 to modulate PGLYRPs in both MoDCs and PIE cells. This is consistent with our previous results demonstrating that BB536 strain is able to activate the TLR2-NF-kB signaling pathways more effectively than M-16V strain [35]. As we discussed earlier, our findings indicate that TLR2 would have a negative regulatory role in the expression of PGLYRPs in porcine IECs. This is in agreement with the observation that the bifidobacteria strain (BB536) with the higher capacity to stimulate TLR2 [35] was the strain with the lowest ability to modulate PGLYRPs expression in PIE cells. In addition, the suppression of TLR2 expression in PIE cells by RNA interference significantly increased the capacity of M-16V to up-regulate the expression of PGLYRP-1, -2, and -3. Then, whether paraimmunobiotic *B. breve* M-16V and *B. longum* subsp. *longum* BB536 has a different ability to modulate the expression of PGLYRPs in vivo, improve resistance to infection and/or reduce the severity of intestinal inflammatory diseases is an important topic for future investigations. In addition, comparatively assessing the immunomodulatory capacity of viable and non-viable bifidobacteria in vivo focusing on the variations of their effects according to the dose administered is an open question, which we propose to address in the near future with the aim of advancing in the possible practical applications of paraimmunobiotics. 

## 5. Conclusions

We showed in this work for the first time the expression patterns of PGLYRP-1, PGLYRP-2, PGLYRP-3, and PGLYRP-4 in porcine IECs and APCs, and the modulation of their expression by PRRs ligands. The analysis of the expression of PGLYRP members in porcine tissues indicates that they would be involved in host defense against pathogens and in the maintenance of normal microbiota in the porcine host, as it has been described for other species. Our results shed light on the impact of some PRRs in the expression of PGLYRPs in porcine APCs and IECs, which contributes not only to the understanding of the factors that regulate PGLYRPs expression in the porcine gut but in addition provide the scientific basis for the use of our in vitro porcine systems as suitable models for the study of PGLYRPs´ immunobiology. We also reported here for the first time that PGLYRPs expression in porcine in APCs and IECs can be differentially modulated by paraimmunobiotic bifidobacteria. These results provide evidence for the use of paraimmunobiotic bifidobacteria as an alternative for the improvement of resistance to intestinal infections or as therapeutic tools for the reduction of the severity of inflammatory damage in diseases in which a role of PGLYRPs-microbe interaction has been demonstrated.

## Supplementary Materials

The following are available online. All the data related to this manuscript are provided with graphs and figures as well as supplementary information.
