# Supplementary material for: Paraimmunobiotic Bifidobacteria Modulate the Expression Patterns of Peptidoglycan Recognition Proteins in Porcine Intestinal Epitheliocytes and Antigen Presenting Cells
Source: Cells. 2019 Aug 14;8(8):891. doi: 10.3390/cells8080891 (PMC6721749; doi:10.3390/cells8080891)
Supplement: Supplementary file 1 [file cells-08-00891-s001.pdf]

Supplementary materials

Paraimmunobiotic bifidobacteria modulate the expression patterns of peptidoglycan recognition proteins in porcine intestinal epitheliocytes and antigen presenting cells

Hikaru Iida[1, 2†], Masanori Tohno[3†], Md. Aminul Islam[1, 2,12 a †], Nana Sato[1, 2], Hisakazu Kobayashi[1, 2], Leonardo Albarracin[1, 11], AKM Humayun Kober[1,2a], Wakako Ikeda-Ohtsubo[1,2], Yoshihito Suda[4], Hisashi Aso[2,5], Tomonori Nochi[5, 6], Ayako Miyazaki[7], Hirohide Uenishi[8], Noriyuki Iwabuchi[9], Jin-zhong Xiao[10], Julio Villena[1,11*] and Haruki Kitazawa[1, 2*]

[1] Food and Feed Immunology Group, Laboratory of Animal Products Chemistry, Graduate School of Agricultural Science, Tohoku University, Sendai, Japan.
[2] Livestock Immunology Unit, International Education and Research Center for Food Agricultural Immunology (CFAI), Graduate School of Agricultural Science, Tohoku University, Sendai, Japan.
[3] Central Region Agricultural Research Centre, National Agriculture and Food Research Organization, Nasushiobara, Japan.
[4] Department of Food, Agriculture and Environment, Miyagi University, Sendai, Japan.
[5] Cell Biology Laboratory, Graduate School of Agricultural Science, Tohoku University, Sendai, Japan.
[6] Infection Immunology Unit, International Education and Research Center for Food Agricultural Immunology (CFAI), Graduate School of Agricultural Science, Tohoku University, Sendai, Japan.
[7] Viral Diseases and Epidemiology Research Division, National Institute of Animal Health, NARO, Tsukuba, Japan

[8] Animal Bioregulation Unit, Division of Animal Sciences, Institute of Agrobiological Sciences, National Agriculture and Food Research Organization (NARO), Tsukuba, Ibaraki, Japan.

[9] Food Science and Technology Institute, Morinaga Milk Industry Co. Ltd, Zama, Kanagawa, Japan.
[10] Next Generation Science Institute, Morinaga Milk Industry Co. Ltd, Zama, Kanagawa, Japan.
[11] Laboratory of Immunobiotechnology, Reference Centre for Lactobacilli, (CERELA-CONICET), Tucuman, Argentina.
[12] Department of Medicine, Faculty of Veterinary Science, Bangladesh Agricultural University, Mymensingh-2202, Bangladesh.

[†] These authors have contributed equally to this work
[a] JSPS Postdoctoral Fellow

* **Correspondence:** haruki.kitazawa.c7@tohoku.ac.jp; Tel. +81-22-757-4372
jcvillena@cerela.org.ar; Tel: +54-381-4310465

Supplementary Figures

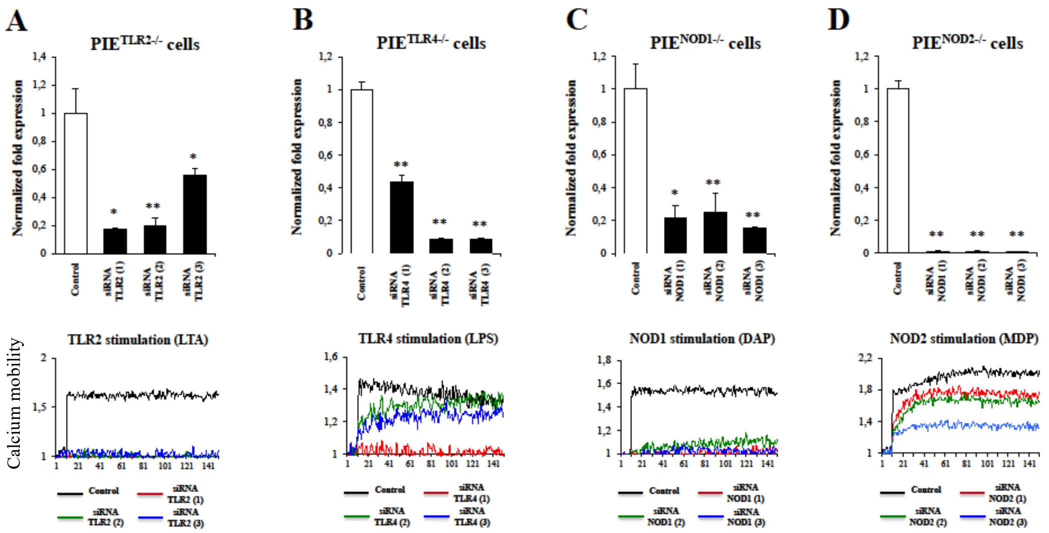

Supl. Figure 1A-D. Porcine TLR2, TLR4, NOD1 and NOD2 expression in PIE cells transfected with siRNA of TLR2, TLR4, NOD1 and NOD2. Each mRNA expression level was normalized by that of the porcine β-actin mRNA, and the normalized fold expression was determined in comparison to the TLR2, TLR4, NOD1 and NOD2 mRNA level of non-transfected PIE cells. Values represent the means and error bars indicate the standard deviation. *p<0.05, **p<0.01, ***p<0.001 against non-transfected PIE cells. The graphs of lower panel illustrate the effect of PRR-ligand stimulation on calcium effluxes in PIE cells. The induction of intracellular calcium mobilization after stimulations with LTA（TLR2 ligand, LPS（TLR4 ligand, Tri-DAP（NOD1 ligand and MDP（NOD2 ligand) were evaluated by recording fluorescence intensity.

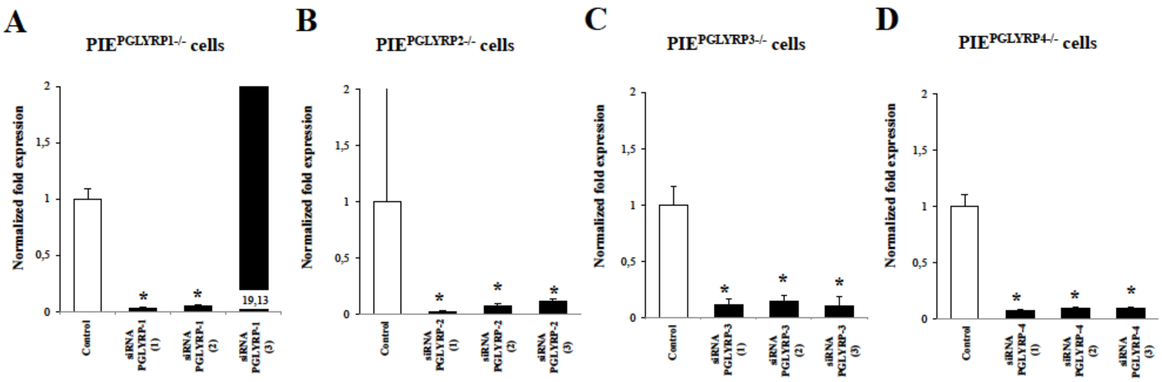

Supl. Figure 2A-D. Porcine PGLYRP-1, PGLYRP-2, PGLYRP-3, and PGLYRP-4 expression in the PIE cells transfected with siRNA of PGLYRP-1, PGLYRP-2, PGLYRP-3, and PGLYRP-4. The porcine PGLYRP mRNA expression level was normalized by that of the porcine β-actin mRNA, and the normalized fold expression was determined in comparison to the PGLYRP mRNA level of non-stimulated PIE cells. Values represent the means and error bars indicate the standard deviation. **p*<0.05, ***p*<0.01, ****p*<0.001 against non-transfected PIE cells.

Supplementary Tables

Supplementary Table 1. Sequences of DNA plasmid of PGLYRP mRNAs used for qRT-PCR study.

Target gene	Plasmid DNA sequence
Porcine PGLYRP-1	CCATCAGGGCGGCCCAAAGTCTGCTGGCTTGTGGGGTGGCTCT<br>GGGAGTCCTGATGCCCAACTACGATGTCAAAGGACACCGGGA<br>TGTGCAGCCAACGCTTTCTCCAGGTGACCAGCTGTACGAAATC<br>ATCCAGAAATGGCCACACTACCGCGCCTGAGCCCCGCTCCTCA<br>CACTGGCTTCCCACCCCCACCCAACCCAT
Porcine PGLYRP-2	CTCCCGCACTGCGCTGTGCGCGCCGGCCTCCTGCAGCCAGACT<br>ATGCGCTGCTCGGCCACCGCCAGCTCGTGCGCACTGACTGCCC<br>TGGCGACGCGCTCTTCAACATGCTGCGCACCTGGCCACGCTTC<br>AACATGAATGTGAAACCAAGAACTGCCAGGAGGGCCTCAGGG<br>AGATCCAAAAGGAGGCTACCTCTAATGAT
Porcine PGLYRP-3	ATGAAGGAGGGCCACCTGTCCCCCAGGTATATTCAGCCACTGC<br>TTTTGAAAGAAGAGAGCTGCCTGGTCCCTCAACAGCCAGTGAT<br>GCCCGGGAAAGCTTGCCCCAACATCATCACAAGGTCAACTTGG<br>GAAGCCAGACAGACACACTGCCCTACAATGAACCTTCCAGCC<br>AAATACGTCATCATCATTCACACCGCCGG
Porcine PGLYRP-4	TGTCTGGAAGGGCCACCTGTCCGCCATGTATGTCCAGCCGCTT<br>CTTGTGAAAGGCGAGAGCTGCCTGAACCCTCGGCAGAATGCA<br>AGTCACAAGGAAGCTTGCCCCCTCATTGTCCTGCGGTCTTCTTG<br>GGAGGCCAGGGGGACCCACTGCCCCAAGATGAGCCTGCGGGC<br>TAAGTACGTCATCATCAGCCACACCACTG
Porcine β-actin	TTCCGCTGCCCCGAGGCGCTCTTCCAGCCCTCCTTCCTGGGCAT<br>GGAATCCTGCGGCATCCACGAAACTACCTTCAACTCCATCATG<br>AAGTGCGACGTCGACATCCGCAAGGACCTCTACGCCAACACG<br>GTGCTGTCGGGTGGCACCACCATGTACCCAGGCATCGCCGACA<br>GGATGCAGAAGGAGATCACGGCCCTGGC

Supplementary Table 2. Primer sequences used for house-keeping gene β-actin and TLR2, TLR4, NOD1 and NOD2

Target gene	Sense	Antisense
Porcine β-actin	CATCACCATCGGCAACGA	GCGTAGAGGTCCTTCCTGATGT
Porcine TLR2	ACATGAAGATGATGTGGGCC	TAGGAGTCCTGCTCACTGTA
Porcine TLR4	CTCTGCCTTCACTACAGAGA	CTGAGTCGTCTCCAGAAGAT
Porcine NOD1	CTGTCGTCAACACCGATCCA	CCAGTTGGTGACGCAGCTT
Porcine NOD2	GAGCGCATCCTCTTAACTTTCG	ACGCTCGTGATCCGTGAAC

Supplementary Table 3. Concentrations for the specific receptor-ligands used.

Ligand	Receptor	Origin	Concentration
Zymosan	TLR2		4 µg/mL
LTA		*B. subtillis*	8 µg/mL
LPS	TLR4	*E. coli*	1 µg/mL
Flagelin	TLR5	*S. typhimurium*	0.8 µg/mL
Imiquimod	TLR7		4 µg/mL
CL075	TLR7/8		0.2 µg/mL
ODN2006	TLR9	Human	20 µg/mL
Tri-DAP	NOD1		2 µg/mL
MDP	NOD2		10 µg/mL

Supplementary Table 4. Primer sequences for siRNAs used for knockdown of different target genes.

Target gene	Sequence (5' to 3')
TLR2(1)	F: CCAGGAACUUGAGAUUGGUGCCUCA<br>R: UGAGGCACCAAUCUCAAGUUCCUGG
TLR2(2)	F: CAGAUGCCUCCUUUCUACCCAUGUU<br>R: AACAUGGGUAGAAAGGAGGCAUCUG
TLR2(3)	F: GAGAACUUUGUGAAGAGAGCGAGUGGU<br>R: ACCACUCGCUCUUCACAAAGUUCUC
TLR4(1)	F: GAGCUUAAUGUGGCUCACAAUCAUA<br>R: UAUGAUUGUGAGCCACAUUAAGCUC
TLR4(2)	F: CAGUGGAAAUCACUUGAGCUUUAAA<br>R: UUUAAAGCUCAAGUGAUUUCCACUG
TLR4(3)	F: GAAAGCACCUAUGACGCCUUUGUUA<br>R: UAACAAAGGCGUCAUAGGUGCUUUC
NOD1(1)	F: CATCTCACCCTTCAGGCCTTCTTTG<br>R: CAAAGAAGGCCTGAAGGGTGAGATG
NOD1(2)	F: CCTTCAAGAACAAGGACCACTTTCA<br>R: TGAAAGTGGTCCTTGTTCTTGAAGG
NOD1(3)	F: TCACGGTCATCAGACTCAGTGTAAA<br>R: TTTACACTGAGTCTGATGACCGTGA
NOD2(1)	F: CAGAGAATCTTTGCCTAGAAGAAAT<br>R: ATTTCTTCTAGGCAAAGATTCTCTG
NOD2(2)	F: CGTCGACAGTGAGGCTGTTTCTCTT<br>R: AAGAGAAACAGCCTCACTGTCGACG
NOD2(3)	F: CCTTGAGGGACAATCAGAGCTTGAA<br>R: TTCAAGCTCTGATTGTCCCTCAAGG

F, Forward and R, Reverse

Supplementary Table 5. Primer sequences for siRNAs used for knockdown of four PGLYRP genes.

Target gene	Sequence (5' to 3')
PGLYRP-1(1)	F: GGG CUA CAA CUU CCU GAU CGG AGA A<br>R: UUC UCC GAU CAG GAA GUU GUA GCC C
PGLYRP-1(2)	F: CGG AGA AGA CGG GCU UGU GUA UGA A<br>R: UUC AUA CAC AAG CCC GUC UUC UCC G
PGLYRP-1(3)	F: UCC UGA UGC CCA ACU ACG AUG UCA A<br>R: UUG ACA UCG UAG UUG GGC AUC AGG A
PGLYRP-2(1)	F: CCU GUU GAU CCU GUA UGG AUU GCU U<br>R: AAG CAA UCC AUA CAG GAU CAA CAG G
PGLYRP-2(2)	F: UGA GGC CGG CCA UAU UGC AUC UAU G<br>R: CAU AGA UGC AAU AUG GCC GGC CUC A
PGLYRP-2(3)	F: CAC UUG GGU UCU UGU AUA UAC AUC A<br>R: UGA UGU AUA UAC AAG AAC CCA AGU G
PGLYRP-3(1)	F: CCU AUG UCA UUG UGC ACC AGC UCA U<br>R: AUG AGC UGG UGC ACA AUG ACA UAG G
PGLYRP-3(2)	F: CAA GGA ACU UCU GUG ACA UCG GAU A<br>R: UAU CCG AUG UCA CAG AAG UUC CUU G
PGLYRP-3(3)	F: CAA CAU CAU UAA GAC UUG GCC UCA U<br>R: AUG AGG CCA AGU CUU AAU GAU GUU G
PGLYRP-4(1)	F: CCA UGU AUG UCC AGC CGC UUC UUG U<br>R: ACA AGA AGC GGC UGG ACA UAC AUG G
PGLYRP-4(2)	F: CAU GGA CAA AUU GGA CUC GUG UGA U<br>R: AUC ACA CGA GUC CAA UUU GUC CAU G
PGLYRP-4(3)	F: CCU GGG UGG GCU UUG UAC AAC AUC A<br>R: UGA UGU UGU ACA AAG CCC ACC CAG G

F, Forward and R, Reverse
